# Health Monitoring of Bolted Spherical Joint Connection Based on Active Sensing Technique Using Piezoceramic Transducers

**DOI:** 10.3390/s18061727

**Published:** 2018-05-27

**Authors:** Jing Xu, Chenyu Wang, Hongnan Li, Chunwei Zhang, Jiajia Hao, Shuli Fan

**Affiliations:** 1 School of Civil Engineering, Qingdao University of Technology, Qingdao 266033, China; jingxu180@qut.edu.cn (J.X.); zhangchunwei@qtech.edu.cn (C.Z.); haojiajs@hotmail.com (J.H.); 2 State Key Laboratory of Coastal and Offshore Engineering, Dalian University of Technology, Dalian 116024, Liaoning, China; hnli@dlut.edu.cn; 3 Department of Mechanical Engineering, University of Houston, Houston, TX 77004, USA; cwang53@uh.edu; 4 College of Civil Engineering, Shenyang Jianzhu University, Shenyang 110168, China

**Keywords:** space steel structures, bolted spherical joint connection, Lead Zirconate Titanate (PZT), tightness status, active sensing technique, wavelet packet analysis, time reversal method

## Abstract

Bolted spherical joints are widely used to form space steel structures. The stiffness and load capacity of the structures are affected by the looseness of bolted spherical joint connections in the structures. The looseness of the connections, which can be caused by fabrication error, low modeling accuracy, and “false twist” in the installation process, may negatively impact the load capacity of the structure and even lead to severe accidents. Furthermore, it is difficult to detect bolted spherical joint connection looseness from the outside since the bolts connect spheres with rods together from the inside. Active sensing methods are proposed in this paper to monitor the tightness status of the bolted spherical connection using piezoceramic transducers. A triangle-on-triangle offset grid composed of bolted spherical joints and steel tube bars was fabricated as the specimen and was used to validate the active sensing methods. Lead Zirconate Titanate (PZT) patches were used as sensors and actuators to monitor the bolted spherical joint tightness status. One PZT patch mounted on the central bolted sphere at the upper chord was used as an actuator to generate a stress wave. Another PZT patch mounted on the bar was used as a sensor to detect the propagated waves through the bolted spherical connection. The looseness of the connection can impact the energy of the stress wave propagated through the connection. The wavelet packet analysis and time reversal (TR) method were used to quantify the energy of the transmitted signal between the PZT patches by which the tightness status of the connection can be detected. In order to verify the effectiveness, repeatability, and consistency of the proposed methods, the experiments were repeated six times in different bolted spherical connection positions. The experimental results showed that the wavelet packet analysis and TR method are effective in detecting the tightness status of the connections. The proposed active monitoring method using PZT transducers can monitor the tightness levels of bolted spherical joint connections efficiently and shows its potential to guarantee the safety of space steel structures in construction and service.

## 1. Introduction

Space steel structures are widely used in stadiums, theaters, exhibition centers, airport terminals, and many other large-scale structures. Any one of such structures can simultaneously house a large number of occupants. It is very important to guarantee the integrity of these structures. Space steel structures are composed of joints and bars. Bolted spherical joints have been widely used to form space steel structures due to such advantages as good mechanical behavior, flexible connection, and high installation speed. However, there are a variety of issues that arise during the design and installation process of the bolted spherical joints, including low modeling accuracy, joint manufacturing error, and the “false tightening” phenomenon. The issues are difficult to avoid, and may lead to connection looseness and structural failure if not resolved in time [1]. Furthermore, it is difficult to detect bolted spherical joint connection looseness from the outside since the bolts connect spheres with rods together from the inside [2]. Therefore, it is very important to monitor the tightness of the bolted spherical joint connections in real time to guarantee the safety of structures.

Currently, most research on joint connection monitoring is focused on individual bolt connection [3,4,5] or flange joint connections in transmission towers and beam-column joint connections in steel frame and steel truss structures [6,7,8]. As for space steel structures, the structural damages are mostly simulated by decreasing stiffness of rods and few studies focused on the bolted spherical joint connection performance [9,10,11]. In the German Mero system, the steel pipes are opened to monitor if high-strength bolts are fully tightened [12]. As a result, the stress on the cross section is concentrated and the intensity on the net section is weakened, which leads to a reduction in capacity and stiffness of steel-pipe bars. In the Dalian TV tower project, the endoscopes were inserted in the holes of nuts in order to identify whether the high-strength bolts were in the right position [13]. This method requires high operation skills and greatly increases construction cost. It is not fit for the popularization and application in the practice. In fact, the probability of joint looseness is significantly higher than that of damage in bars for space steel structures [14]. Therefore, it is very important to find a reliable, nondestructive, real-time, and simple method for monitoring the looseness status of bolted spherical connections.

Structure Health Monitoring (SHM) is the process of identifying the damage and evaluating the health status of the structure in real time [15,16]. The SHM methods for space steel structures are often based on the measurements of the structural modal parameters, such as natural frequencies [17,18], mode shapes [19], and modal damping [20]. These methods measure the structural modal parameters when the structure is subjected to environmental wind loads or manual impact excitation. The additional requirements of the vibration excitation sources limit the practical applications of such methods for monitoring the health condition of space steel structure connections in real time. In recent years, the piezoceramic transducer-enabled active sensing method received much attention in the SHM [21,22,23,24,25]. With its inherent ability to convert between mechanical energy and electrical energy [26,27,28], a piezoceramic transducer has both actuating [29] and sensing capacities [30,31,32,33]. In addition, with its wide bandwidth up to hundreds of megahertz [34,35,36], a piezoceramic transducer is often used to generate a stress wave and also to detect stress waves [37,38]. Lead Zirconate Titanate (PZT) is a commonly-used piezoceramic material due to its strong piezoelectric effect, low cost, and commercial availability [39,40]. PZT transducers can be fabricated into different shapes, such as commonly-used patches, rings, and even spheres [41,42], for a wide range of applications. In PZT-enabled active sensing, at least a sensor-actuator pair is needed, and often distributed PZT transducers are used. During the active sensing process, one PZT transducer is used as an actuator to generate a stress wave, which propagates along a structure of interest, and other PZT transducers on the structure will detect the propagating stress wave [43,44,45]. Damages on a structure, such as a crack or a loosened connection, will further attenuate the propagating stress wave [46,47,48], and therefore, by monitoring the stress wave detected by the PZT sensors, the structural damages can be detected [49,50,51,52].

In the active sensing method, wavelet packet analysis [53,54] and time reversal technique [55,56] are both effective methods for data processing. Wavelet packet transform can provide enough of both frequency resolution and computational effectiveness at the same time in the analysis. Wavelet packet transform was used to assess the damage in the structures [57,58] and to monitor the looseness of the connections in cuplock scaffolding structures [59]. Since the time reversal method was first introduced into ultrasonic fields by Fink [60,61], it was widely used due to the self-focusing and anti-noise properties. In structural health monitoring, the time reversal method was applied in the damage detection and localization of metal and composite plates [62,43]. In the health monitoring of the mechanical connections, the time reversal method was also used to detect the looseness of the connections. The authors proposed a looseness monitoring method for the cuplock connection based on the time reversal method in [63]. Health monitoring methods were developed based on the time reversal method to detect the looseness of bolted joints [64,65]. In this paper, wavelet packet analysis and time reversal methods are proposed to detect the tightness status of the bolted spherical joint connections in space steel structures with PZT transducers. A triangle-on-triangle offset grid with bolt-sphere joints is used as the specimen.

In this paper, a novel application of health monitoring method based on active sensing technique is developed to monitor the tightness status of the bolted spherical connection using piezoceramic transducers. Experiments on a triangle-on-triangle offset grid composed of bolted spherical joints and steel tube bars were conducted to verify the efficiency of the proposed method. The rest of the paper is organized as follows. The detection principle for bolt-sphere joint tightness is presented in Section 2. Section 3 describes the experimental setup. Section 4 reports the experiments that were conducted to validate the relationship between the degree of tightness and the peak amplitude of focused signal based on the time reversal method and the looseness index based on wavelet packet analysis, respectively. Section 5 concludes the paper.

## 2. The Principle for Monitoring the Tightness of Bolt-Sphere Connection Based on Active Sensing Technique

### 2.1. Bolted Spherical Joint Structure

**Figure 1:**
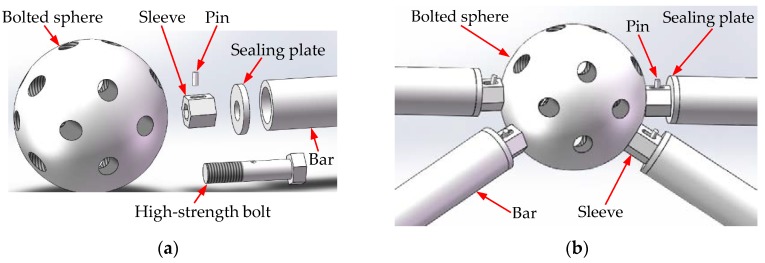
Diagram of bolt-sphere joint structure: (**a**) Bolt-sphere joint parts; (**b**) Bolt-sphere joint.

A space steel structure often consists of hundreds of bolted spherical joints. As shown in Figure 1, there are mainly five parts in a bolted spherical joint: a bolted sphere, a high-strength bolt, a sleeve, a pin, and a bar with a sealing plate. The installation process is as follows. With the sleeve being screwed, the pin moves from the bar side to the joint side. At the same time, the high-strength bolt, which is driven by the moving pin, connects the bar, the bolted sphere, and the sleeve tightly together from the inside. The position of the high-strength bolt cannot be observed from the outside, which makes it difficult to monitor the tightness status of the bolted spherical joints.

### 2.2. Basic Principle of Bolt-Sphere Connection Monitoring Based on Active Sensing Technique

**Figure 2:**
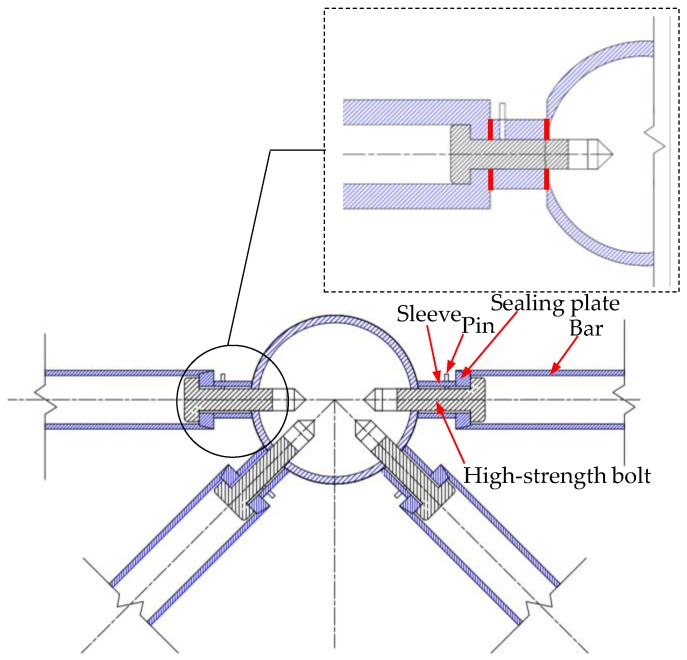
Cross section of bolted spherical joint connection.

The cross-sectional view of the bolted spherical joint connection is shown in Figure 2. Two thick, red lines in the detail drawing represent two contact interfaces. One is the contact interface between the bolted sphere and the sleeve. Another is the contact interface between the sleeve and the bar. According to contact mechanics, the contact area of two elastic bodies is proportional to the square root of normal force on the contact interface [66]. Moreover, as the connection tightness increases, normal force increases correspondingly. Therefore, the tightness status of the connection can be characterized by the contact area [66]. The tighter the connection is, the greater the normal force on the contact interface will be and the larger the contact area will be.

PZT1 is surface-bonded on the central bolted sphere at the upper chord and acts as an actuator to generate stress waves. PZT2 is surface-bonded on the end of the bar, which is near the bolt sphere, and acts as a sensor for receiving stress waves. The stress wave propagation is as follows: At first, upon receiving an input signal, PZT1 generates a stress wave. Then, the stress wave propagates through the contact interface between the bolted sphere and the sleeve. After that, the stress wave continues to propagate through the contact interface between the sleeve and the bar. Finally, the stress wave is detected by PZT2. In this process, the tightness of the joint connection will directly impact the stress wave signal intensity received by PZT patches.

**Figure 3:**
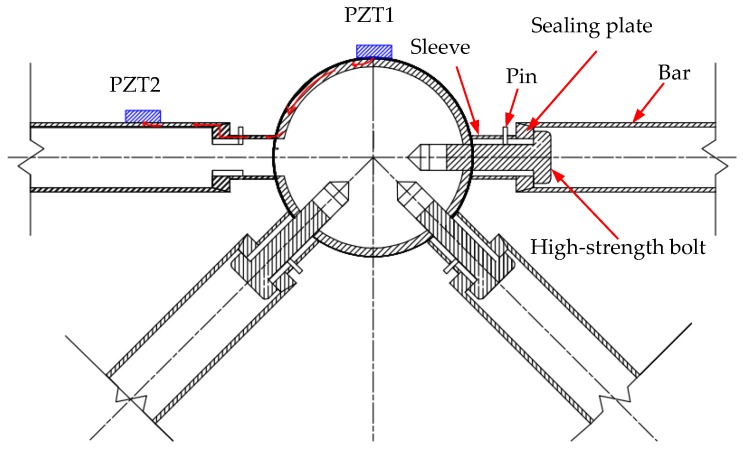
Energy transmission in bolted spherical joint connection.

Stress wave propagation is essentially the process of energy transmission, just as shown in Figure 3. Figure 3 shows the direction of energy transmission. The direction of energy transmission is as follows: At first, upon receiving an input signal, PZT1 generates a stress wave. Then, along the wall of the bolted spherical joint, the stress wave energy propagates through the contact interface between the bolted sphere and the sleeve. After that, along the wall of the sleeve, the stress wave energy continues to propagate through the contact interface between the sleeve and the bar. Finally, along the wall of the bar, the stress wave energy continues to propagate until it is detected by PZT2. There is some energy loss when the wave propagates through interior materials and contact interfaces. If structural material has no change, the amount of wave energy loss within the material is certain. At this time, the transmitted energy can be decided only on the contact area. That is, the larger the contact area is, the more the transmitted energy is. Moreover, the tighter the connection is, the larger the true contact area is. It can be concluded that the tighter the connection is, the more the transmitted energy is and the stronger the stress wave signals are. In the proposed approach, the stress wave signals received by PZT patches are used to monitor the tightness of bolted spherical joint connections.

### 2.3. Basic Principle of Bolt-Sphere Connection Monitoring Based on Wavelet Packet Technology

Wavelet packet analysis is an extension of wavelet analysis. It can further decompose the high-frequency components unprocessed by the wavelet transform into independent frequency bands. This decomposition has the advantages of no omission, no residue, and orthogonality. Wavelet packet analysis is a more precise method for signal processing.

If a signal X is decomposed by the *n*—layer wavelet packet into 2*^n^* frequency bands, X can be expressed as $\left\{X_{1},X_{2},\cdots ,X_{j},\cdots ,X_{2^{n}}\right\}$, $X_{j}$ can be further expressed as:

(1) $$X_{j}=\left[x_{j,1},x_{j,2},\cdots ,x_{j,m}\right]\;\left(j=1,2,\cdots ,2^{n}\right)$$

where j stands for the jth frequency band and m indicates the total number of samples.

The connection is divided into p tightness status. When the connection is at the ith tightness status, the energy vector $E_{i}$ is as follows:

(2) $$E_{i}=\left[E_{i,1},E_{i,2},\cdots ,E_{i,2^{n}}\right]\;\left(i=1,2,\cdots ,p\right)$$

where $E_{i,j}$ can be further expressed by $E_{i,j}=||X_{j}||{}_{2}^{2}=x_{j,1}^{2}+x_{j,2}^{2}+\cdots +x_{j,m}^{2}$.

The looseness index I can be defined by the root mean square difference (RMSD) of the energy $E_{i,j}$. It represents the tightness status of the connection.

As the bolted spherical joint is tightened to reach the required preload level (healthy status), the corresponding energy vector can be expressed as:

(3) $$E_{t}=\left[E_{t,1},E_{t,2},\cdots ,E_{t,2^{n}}\right]$$

Based on the Formulas (2) and (3), when the connection is at the ith tightness status, the looseness index I(i) can be expressed as:

(4) $$I\left(i\right)=\sqrt{\overset{2^{n}}{\underset{j=1}{\sum }}{\left(E_{i,j}-E_{t,j}\right)}^{2}/\overset{2^{n}}{\underset{j=1}{\sum }}E_{t,j}^{2}}\;\left(i=1,2,\cdots ,p\right)$$

where p is the total number of tightness status and j is the frequency band number.

The looseness index I(i) represents the relative loss of energy transmitted by the bolted spherical joint at the ith tightness status. The smaller the loosening index I(i) is, the less the energy loss is and the tighter the joint is. If the loosening index I(i) equals to zero, the connection is at the tightest status.

### 2.4. Basic Principle of Bolt-Sphere Connection Monitoring Based on Time Reversal Method

The time reversal is a reverse operation on the received signal in the time domain. It is a process of reversing acoustic or electromagnetic wave signals from the object and then sending it back. The reversal signals have the characteristics of spatial and temporal focusing properties. These properties can solve the multiscattering problem of acoustic detection in complex environments.

In this paper, the time reversal method has three steps.

In the first step, a pulse input is applied to PZT1. Then PZT1 (acting as an actuator) generates a stress wave. The generated stress wave propagates through the bolted spherical joint connection. At last, the stress wave is captured by PZT2 (acting as a sensor). The process can be expressed as follows:

(5) y(t)=x(t)⊗h(t)

where y(t) is the received signal by PZT2, x(t) is the pulse input, h(t) represents impulse response function, and ⊗ denotes the convolution operation.

In the second step, the signal received is reversed in time domain, which is the time reversal process and this process can be expressed as follows:

(6) y(−t)=x(−t)⊗h(−t)

In the third step, the reversed signal y(−t) is applied to PZT2, which acts as an actuator and retransmits the signal back to PZT1. PZT1, acting as a sensor, receives the focusing signal named $y_{F}\left(t\right)$ that can be expressed in the following equation:

(7) $$y_{F}\left(t\right)=y\left(-t\right)⊗h\left(t\right)=x\left(-t\right)⊗\left[h\left(t\right)⊗h\left(-t\right)\right]$$

According to the principle of convolution and correlation, h(t)=h(−t). Meanwhile, the pulse input signals applied in the experiment are time reversal symmetric, meaning that x(t)=x(−t). Thus, the focusing $y_{F}\left(t\right)$ is a convolution of the input signal and the autocorrelation function and can be expressed as follows:

(8) $$y_{F}\left(t\right)=x\left(t\right)⊗\left[h\left(t\right)⊙h\left(t\right)\right]$$

where ⊙ denotes the correlation operation. The autocorrelation function [h(t)⊙h(t)] is an even function which is often called the time reversal operator. It can achieve the maximum value $\int_{-\infty }^{+\infty }h^{2}\left(\tau \right)d\tau$ when t=0. This maximum value is equal to the energy of the impulse response function of the structure between PZT1 and PZT2.

For the reason that the input signal is supposed as a pulse signal, it can be written as x(t)=βδ(t). Equation (8) will be:

(9) $$y_{F}\left(t\right)=\int_{-\infty }^{+\infty }h\left(\tau \right)h\left(\tau -t\right)d\tau$$

where β is the amplitude of x(t). Thus, there is a focused peak with the signal $y_{F}\left(t\right)$ received by PZT1. The amplitude of the focused peak is proportional to the energy of the impulse response function according to Equation (8). According to Section 2.2, as the bolted spherical joint tightness increases, the energy of the impulse response increases correspondingly. Therefore, the peak amplitude of the focused signal is used as an index to monitor the tightness status of the bolted spherical connection.

## 3. Experimental Setup

**Figure 4:**
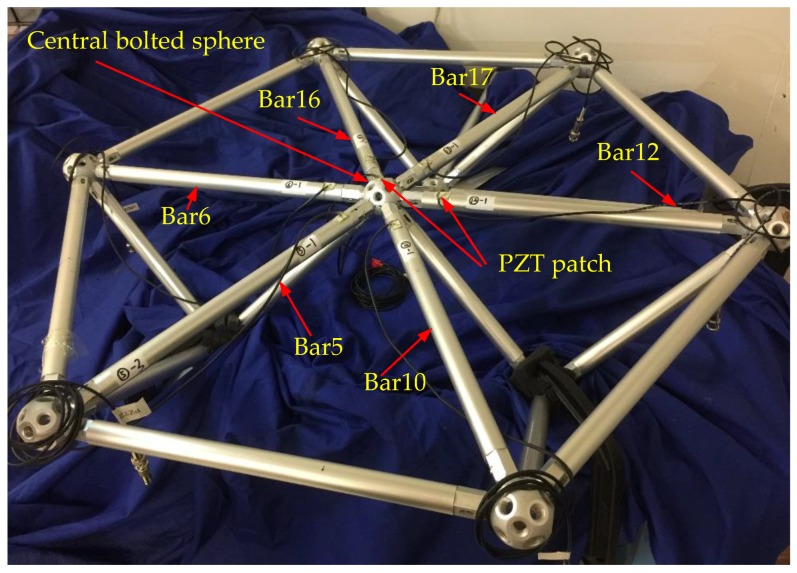
Photo of triangle-on-triangle offset grid model.

**Figure 5:**
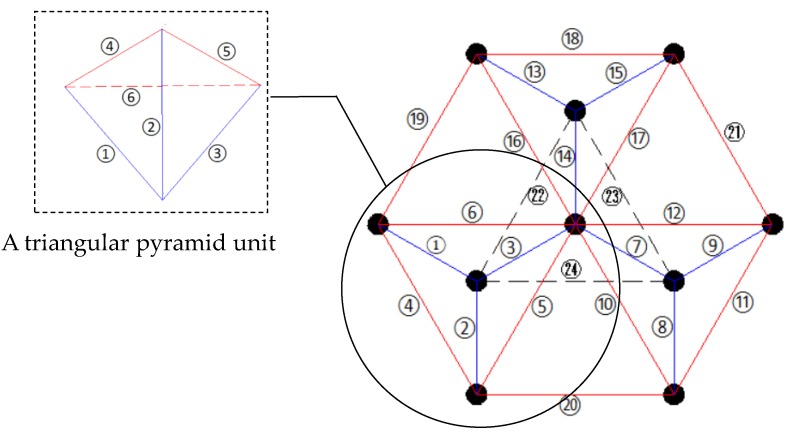
The codes of bar elements in triangle-on-triangle offset grid model.

To simulate the bolted spherical joint connection, a triangle-on-triangle offset grid model was designed and constructed. It was 0.2 m in height and composed of three orthogonal triangular pyramid units, as shown in Figure 4. Figure 5 shows the codes of bar elements. The material properties of the experimental model are shown in Table 1. Dimensions of bar members and bolted spheres were ø22 mm × 6 mm and ø46 mm × 10 mm, respectively. The model was fixed with the base by the clamps to simulate fixed support. To reduce the loss of stress wave, the grid structure was separated from clamps with a thick sponge.

**Figure 6:**
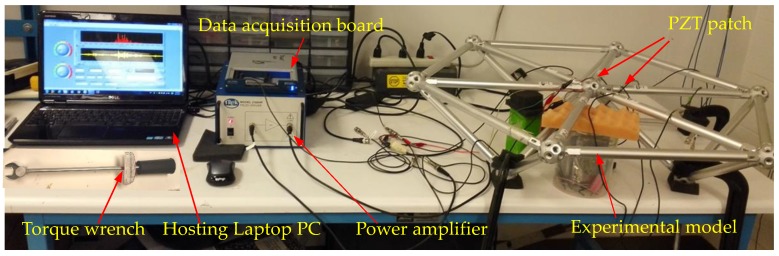
Experimental setup.

**Figure 7:**
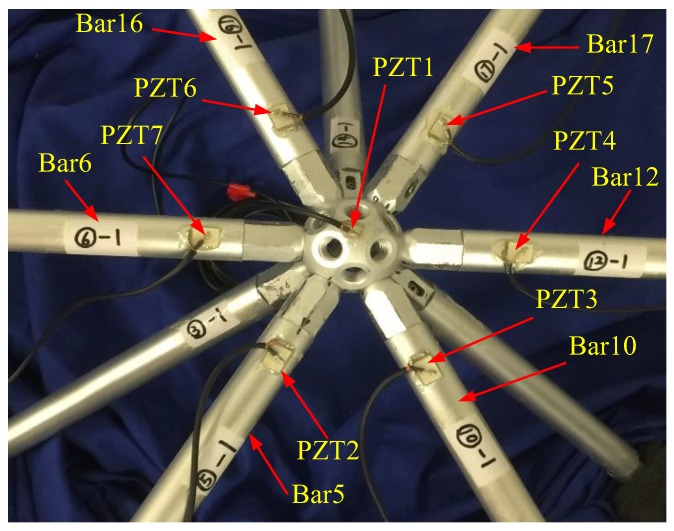
Photo of the surface-bonded PZTs.

Experimental setup mainly included a data acquisition system (NI USB-6363) manufactured by National Instruments Company, a power amplifier (Model 2100HF PIEZO DRIVER) made by TREK Company, a torque wrench made by Craftsman Company, and a laptop, as shown in Figure 6. Nonconductive epoxy was used to mount PZT patches and it also had a protective effect on PZT patches. The placement positions of PZT sensors are shown in Figure 7. From Figure 5, there were six bars connected with the central bolted spherical joint on the upper chord. To verify the consistency of the proposed monitoring method, the experiment was repeated six times to monitor the tightness status of six different connection positions based on active sensing technique using piezoceramic transducers.

## 4. Experimental Procedures and Results

### 4.1. Experimental Procedures

In this experiment, the process of tightening the bolted spherical connection was divided into two steps. In the first step, the sleeve was screwed by hand, which is called the initial tightening. In the second step, the sleeve was gradually screwed by a torque wrench, which is called the final tightening. In this research, the experiments were all started after the initial tightening. Tightening status in the process of final tightening was divided into 8 tightening degrees according to the torque value applied on the sleeve, as shown in Table 2. Different torque values represent different tightness degrees. The greater the torque value was, the tighter the joint connection was. The experiment was repeated six times on six different bars. The codes of the six bars were 5, 10, 12, 17, 16, and 6 (Figure 5). The active sensing technique enabled by PZTs was used to monitor the tightness degrees of bolted spherical connections. The wavelet packet analysis and the TR method were used to analyze the transmitted signal by PZT patches.

### 4.2. Experimental Results and Analysis Based on Wavelet Packet Analysis

**Figure 8:**
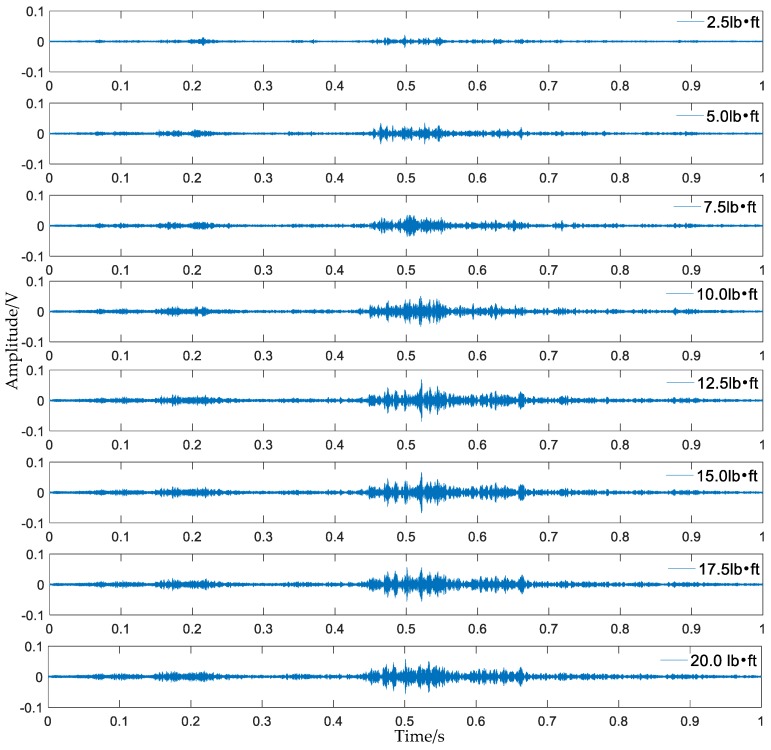
Received signals under eight different torque levels of bar 5.

**Figure 9:**
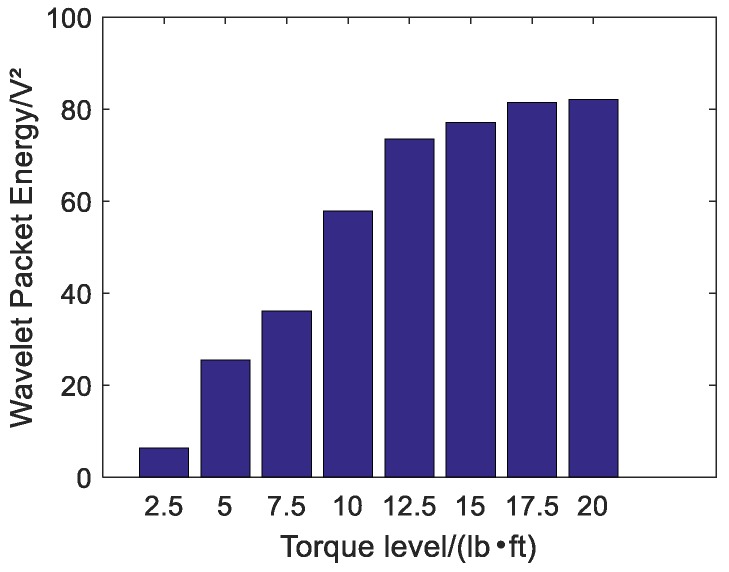
Wavelet packet energy of bar 5 under different torque levels.

**Figure 10:**
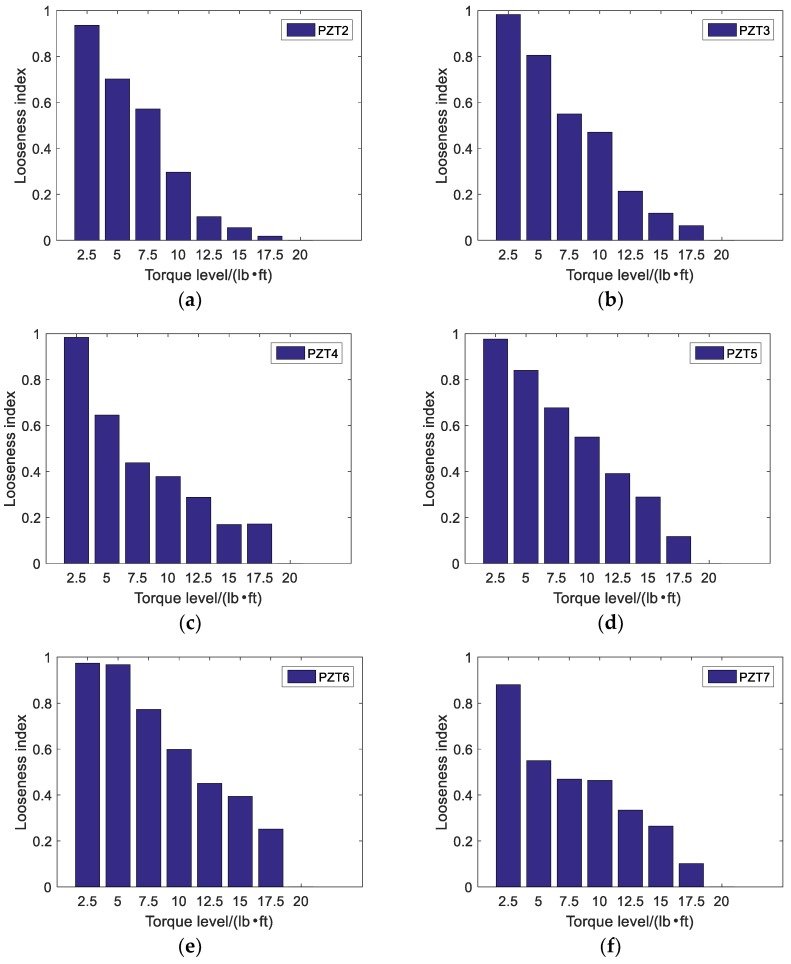
Looseness index at different torque levels: (**a**) Looseness index at PZT2; (**b**) Looseness index at PZT3; (**c**) Looseness index at PZT4; (**d**) Looseness index at PZT5; (**e**) Looseness index at PZT6; (**f**) Looseness index at PZT7.

A repeated swept sine wave was used as an excitation signal for the experiment and parameters of the wave are listed in Table 3. In the experiment, the received stress wave signals were decomposed into 32 signal sets by 5-layer wavelet packet analysis at every tightness status. Taking the bar 5 in Figure 7 as an example, received signals at eight different torque levels are shown in Figure 8. For the ith tightness status, the energy vector $E_{i}$ can be calculated based on Formula (2) and wavelet packet energy is defined as the sum of all elements in vector $E_{i}$. Figure 9 shows the wavelet packet energy of bar 5. The looseness index can be calculated according to Formula (4). Figure 10 shows the looseness index of six bars.

It can be seen from Figure 9 that energy transmitted increased as the torque level increased. From Figure 10, it is clear that the looseness index decreased with the increase in torque level. According to Section 2.2, as the torque level increased, the contact area between connected parts increased and stress wave energy transmitted increased. Thus, the looseness index based on the stress wave energy decreased. As the torque level reached the maximum, the looseness index was about zero and the bolted spherical connection reached its final tightness degree. The result was consistent for the six bars.

### 4.3. Experimental Results and Analysis Based on TR Method

**Figure 11:**
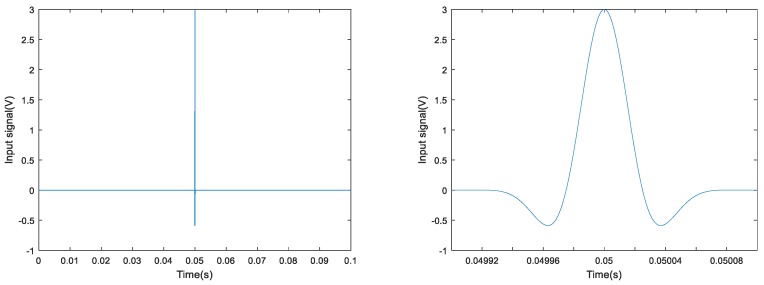
The Gaussian pulse signal.

**Figure 12:**
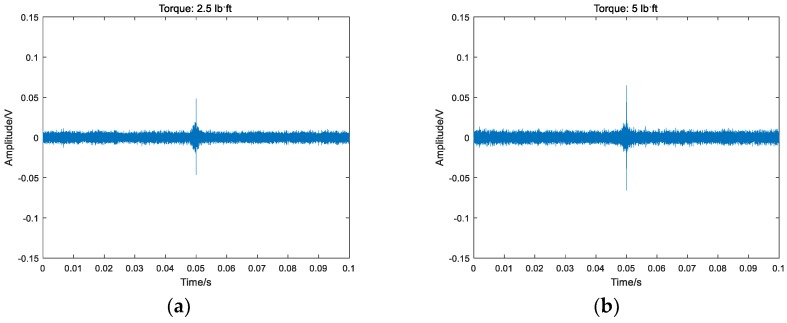

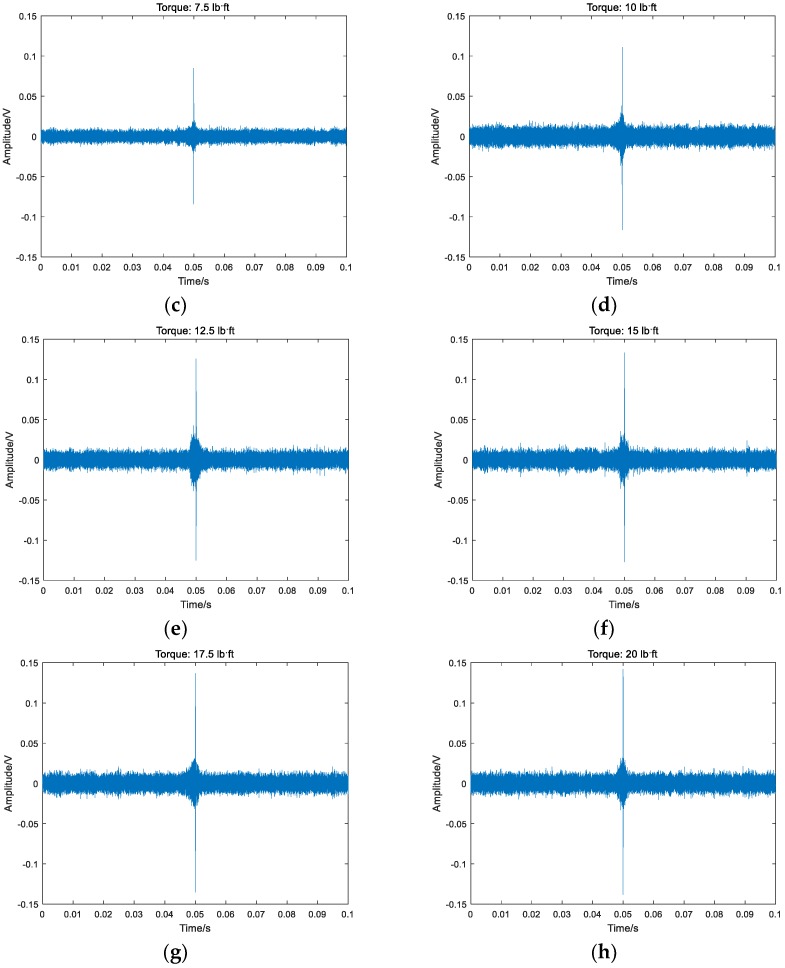
The focused signals based on the time reversal method at different torque levels on bar 5: (**a**) Focused signals at 2.5 lb⋅ft torque; (**b**) Focused signals at 5 lb⋅ft torque; (**c**) Focused signals at 7.5 lb⋅ft torque; (**d**) Focused signals at 10 lb⋅ft torque; (**e**) Focused signals at 12.5 lb⋅ft torque; (**f**) Focused signals at 15 lb⋅ft torque; (**g**) Focused signals at 17.5 lb⋅ft torque; (**h**) Focused signals at 20 lb⋅ft torque.

**Figure 13:**
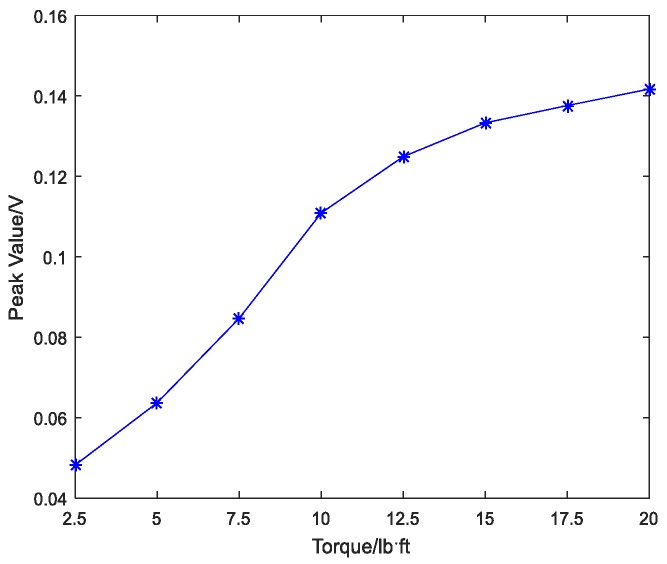
The relationship between the peak value and torque levels on bar 5.

**Figure 14:**
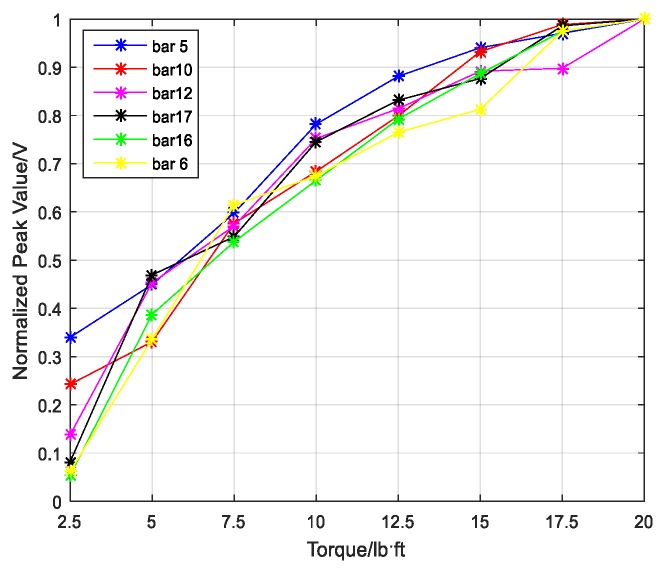
The relationship between normalized peak values and torque levels on six bars.

A Gaussian pulse was used as an excitation signal for the experiment, as shown in Figure 11. The signal’s normalized bandwidth was 0.8 and the central frequency was 10 kHz. The received signal was sampled at the rate of 1 MHz for 0.1 s. Taking the bar 5 in Figure 7 as an example, the focused signals based on the TR method at different torque levels are shown in Figure 12 and the relationship between the peak amplitudes and torque levels is shown in Figure 13. In order to analyze and compare the experimental results for different six bars, the peak values are normalized. Based on the repeated experiments, the relationship between the normalized peak values of the six bars and torque levels is shown in Figure 14.

It is clear that the focused peak value increased with the increase in torque level from Figure 13 and Figure 14. According to Section 2.2, it can be concluded that the tighter the connection is, the more the transmitted energy is and the stronger the stress wave signals are. Thus, the focused peak value based on the stress wave energy increased as the torque level increased. The conclusion is consistent for the six bars connected with the same bolted spherical joint.

**Figure 15:**
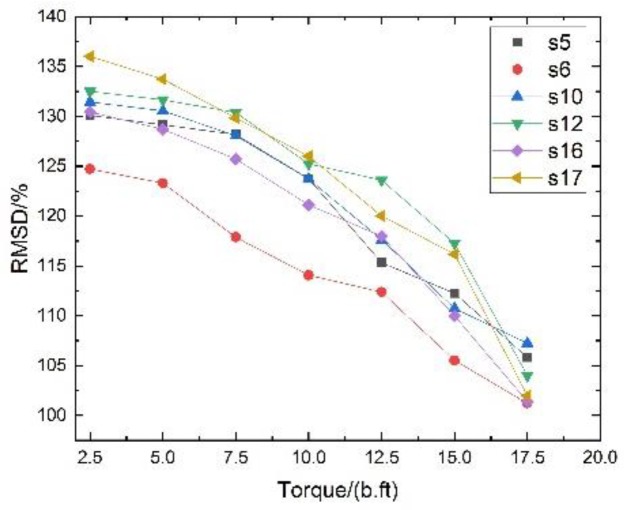
The relationship between the RMSD value and torque levels.

As shown in Figure 15, the root mean square variation (RMSD) index was computed using the Function (4). The focused signals at 20 lb⋅ft torque were chosen as the baseline. The greater RMSD value, the larger the difference between the baseline measurement and the concurrent measurement, indicating the presence of the looser in a structure. The RMSD values for different torque level show a resembling trend with the looseness index computed based on wavelet packet analysis. The RMSD value increased as the torque value applied decreased. It is evident that the RMSD values were able to reflect the loosening status of the bolted spherical joint connection.

The experimental results show that the proposed method based on PZT and TR is effective and stable for monitoring the tightness degree of bolted spherical joint connections. The TR method has excellent repeatability, consistency, and anti-noise ability [56]. For civil engineering structures, the monitoring signals might be affected by factors such as environment, equipment, and human factors because of the complex construction and service conditions. The proposed method based on PZT and TR can improve the accuracy of the monitoring results.

## 5. Conclusions

In this paper, the authors proposed a method based on active sensing technique using piezoceramic transducers to monitor the tightness degree of bolted spherical joint connections. Wavelet packet analysis and time reversal technique were used to process the stress wave signals. Taking a triangle-on-triangle offset grid with bolt-sphere joints as an example, six repeated experiments were conducted on six bars in different positions. The results are consistent. The looseness index calculated by wavelet packet analysis decreases and the focused peak value based on the TR method increases as the tightness level increases. It is clear that the monitoring method of bolted spherical joints based on the piezoceramic sensing technique is feasible and repeatable. The proposed method has the potential to be implemented in the health monitoring of space steel structures connected with bolted spherical joints.

## Figures and Tables

**Table 1:** Material characteristics of experimental model.

Grade of Steel	Elastic Modulus (GPa)	Yield Strengths (MPa)	Steel Density (Kg/m^3^)	Poisson’s Ratio	Elongation
Q345	207	345	7800	0.3	30%

**Table 2:** Torque values at different tightness degrees.

Tightness Degree	First	Second	Third	Fourth	Fifth	Sixth	Seventh	Eighth
Torque (lb⋅ft)	2.5	5	7.5	10	12.5	15	17.5	20

**Table 3:** Parameters of the swept sine wave.

Frequency Bandwidth	Amplitude	Period	Sampling Frequency
1000 Hz–400 kHz	150 V	1 s	2 MHz
